# Absolute quantification of the pretreatment *PML-RARA* transcript defines the relapse risk in acute promyelocytic leukemia

**DOI:** 10.18632/oncotarget.3773

**Published:** 2015-04-18

**Authors:** Francesco Albano, Antonella Zagaria, Luisa Anelli, Nicoletta Coccaro, Giuseppina Tota, Claudia Brunetti, Crescenzio Francesco Minervini, Luciana Impera, Angela Minervini, Angelo Cellamare, Paola Orsini, Cosimo Cumbo, Paola Casieri, Giorgina Specchia

**Affiliations:** ^1^ Department of Emergency and Organ Transplantation (D.E.T.O.), Hematology Section, University of Bari, 70124, Bari, Italy

**Keywords:** acute promyelocytic leukemia, PML-RARA, relapse risk, droplet digital PCR, prognostic factor

## Abstract

In this study we performed absolute quantification of the *PML-RARA* transcript by droplet digital polymerase chain reaction (ddPCR) in 76 newly diagnosed acute promyelocytic leukemia (APL) cases to verify the prognostic impact of the *PML-RARA* initial molecular burden. ddPCR analysis revealed that the amount of *PML-RARA* transcript at diagnosis in the group of patients who relapsed was higher than in that with continuous complete remission (CCR) (272 vs 89.2 *PML-RARA* copies/ng, *p* = 0.0004, respectively). Receiver operating characteristic analysis detected the optimal *PML-RARA* concentration threshold as 209.6 *PML-RARA*/ng (AUC 0.78; *p* < 0.0001) for discriminating between outcomes (CCR versus relapse). Among the 67 APL cases who achieved complete remission after the induction treatment, those with >209.6 *PML-RARA*/ng had a worse relapse-free survival (*p* = 0.0006). At 5-year follow-up, patients with >209.6 *PML-RARA*/ng had a cumulative incidence of relapse of 50.3% whereas 7.5% of the patients with suffered a relapse (*p* < 0.0001). Multivariate analysis identified the amount of *PML-RARA* before induction treatment as the sole independent prognostic factor for APL relapse.

Our results show that the pretreatment *PML-RARA* molecular burden could therefore be used to improve risk stratification in order to develop more individualized treatment regimens for high-risk APL cases.

## INTRODUCTION

Acute promyelocytic leukemia (APL) is associated in almost all cases with chromosomal translocation t(15;17)(q24;q21) involving the *RARA* and *PML* genes at 17q21 and 15q24, respectively, resulting in the fusion transcript *PML-RARA* which encodes for the oncoprotein at the basis of the APL pathogenesis [1]. Combined treatment with anthracyclines, all trans retinoic acid (ATRA) and, more recently, arsenic derivatives, such as arsenic trioxide (ATO), is highly successful, providing long-term remission in the majority of APL patients [2–4]. However, the persistence of resistant leukemic cells after treatment is responsible for relapse in 10–20% of APL patients [3, 5–7]. Until now, the WBC count at diagnosis has been considered the most important prognostic factor in APL, able to better identify those patients at higher risk of relapse [8–10]. Therefore, the presenting WBC is employed to design the consolidation treatment intensity based on risk adapted strategies [4, 10]; this approach aims to reduce the toxicity in APL patients with a low relapse risk, while adopting more intensive targeted therapy in patients at higher risk of relapse. The use of minimal residual disease (MRD) monitoring by real-time quantitative PCR (RT-qPCR) in the clinical management of APL allows prompt detection of molecular relapse before it evolves into frank hematologic relapse [11]. The possibility of intervention while the patient is in molecular instead of hematologic relapse has been demonstrated to improve both the overall and the relapse-free survival of APL patients [11–13]. The optimal frequency of RT-qPCR monitoring in APL is still debated [6, 14–15] but it is reasonable to suppose it may reflect the likelihood of relapse. While in clinical practice the quantification by RT-qPCR of the *PML-RARA* transcript during the postconsolidation phase is a valuable prognostic tool to predict APL relapse [11, 16], the *PML-RARA* molecular burden at diagnosis has not been shown to have a prognostic value, in terms of relapse risk [16]. Droplet digital polymerase chain reaction (ddPCR) is the most accurate and sensitive method to measure the abundance of specific nucleic acids. The purpose of ddPCR is to quantify the absolute number of target present in a sample, implementing PCR data with Poisson statistics [17]. The ddPCR provides a more direct measurement of cDNA copy number and offers a greater precision and reproducibility compared to RT-qPCR [18, 19]. In this study we performed absolute quantification of the *PML-RARA* transcript by ddPCR technology in newly diagnosed APL patients to verify the prognostic impact of the *PML-RARA* initial molecular burden.

## RESULTS

**Figure 1:**
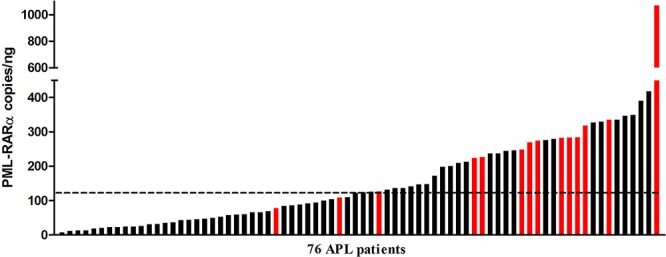
Distribution of the pretreatment *PML-RARA* molecular burden detected by ddPCR The red bars indicate patients who had relapsed. The dashed line indicates the *PML-RARA* copies/ng median value of the entire series (124 copies/ng).

**Figure 2:**
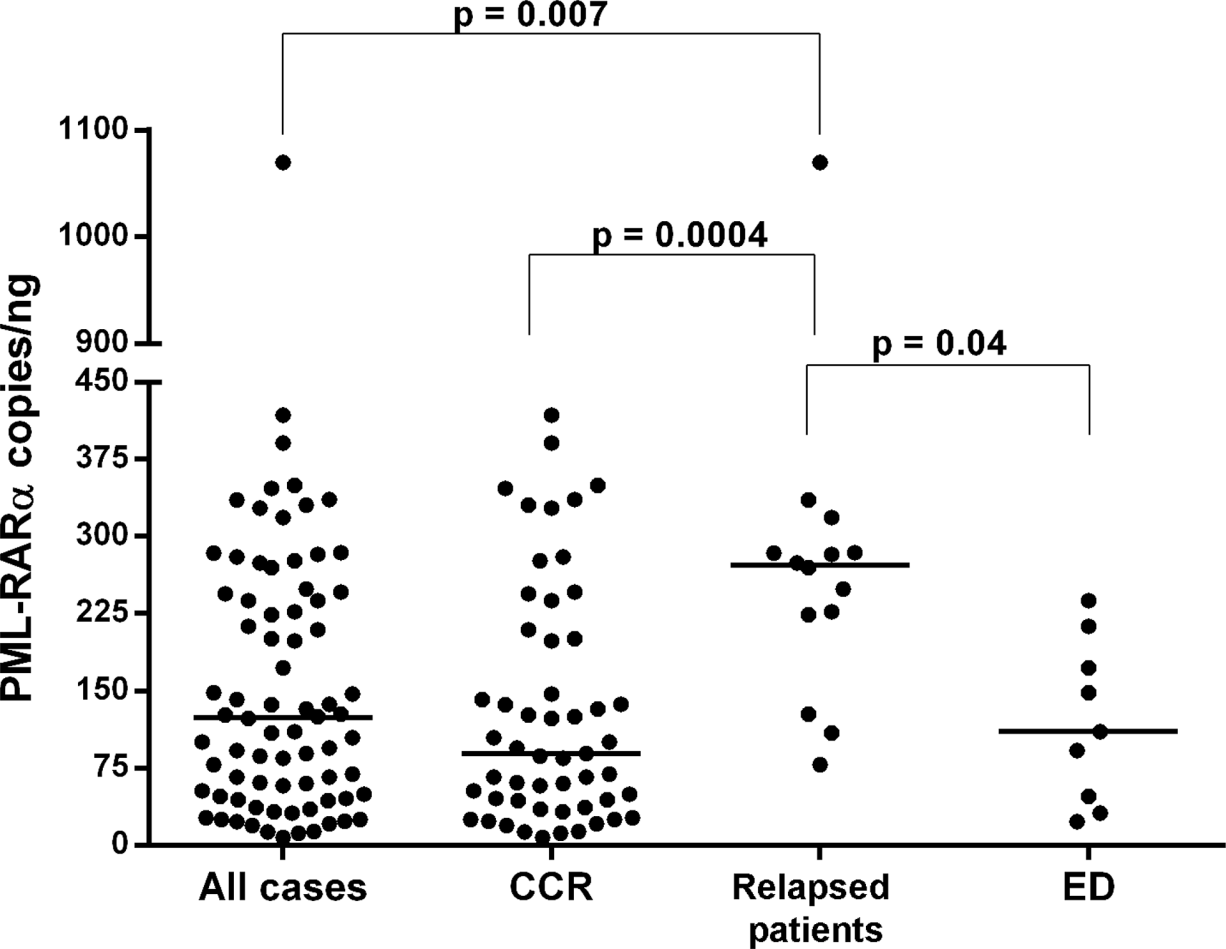
*PML-RARA* copies/ng calculated by ddPCR analysis in APL patients The *PML-RARA* pretreatment molecular burden is reported in the overall cohort, and in the continuous complete remission (CCR), relapsed patients, and early death (ED) patient groups. Each dot represents a patient. The lines indicate the median for each group.

**Figure 3:**
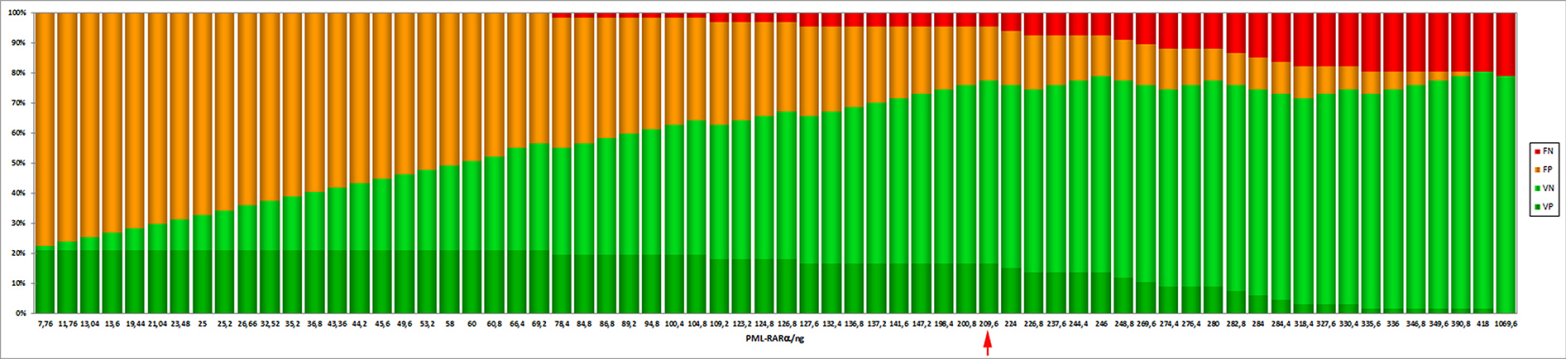
The graph was generated by ROC analysis and shows the count of TP (true positives), TN (true negative), FP (false positives) and FN (false negatives) depending on the chosen threshold value The value corresponding to a concentration of 209.6 copies/ng (red arrow) is the best threshold detected by ROC analysis.

**Figure 4:**
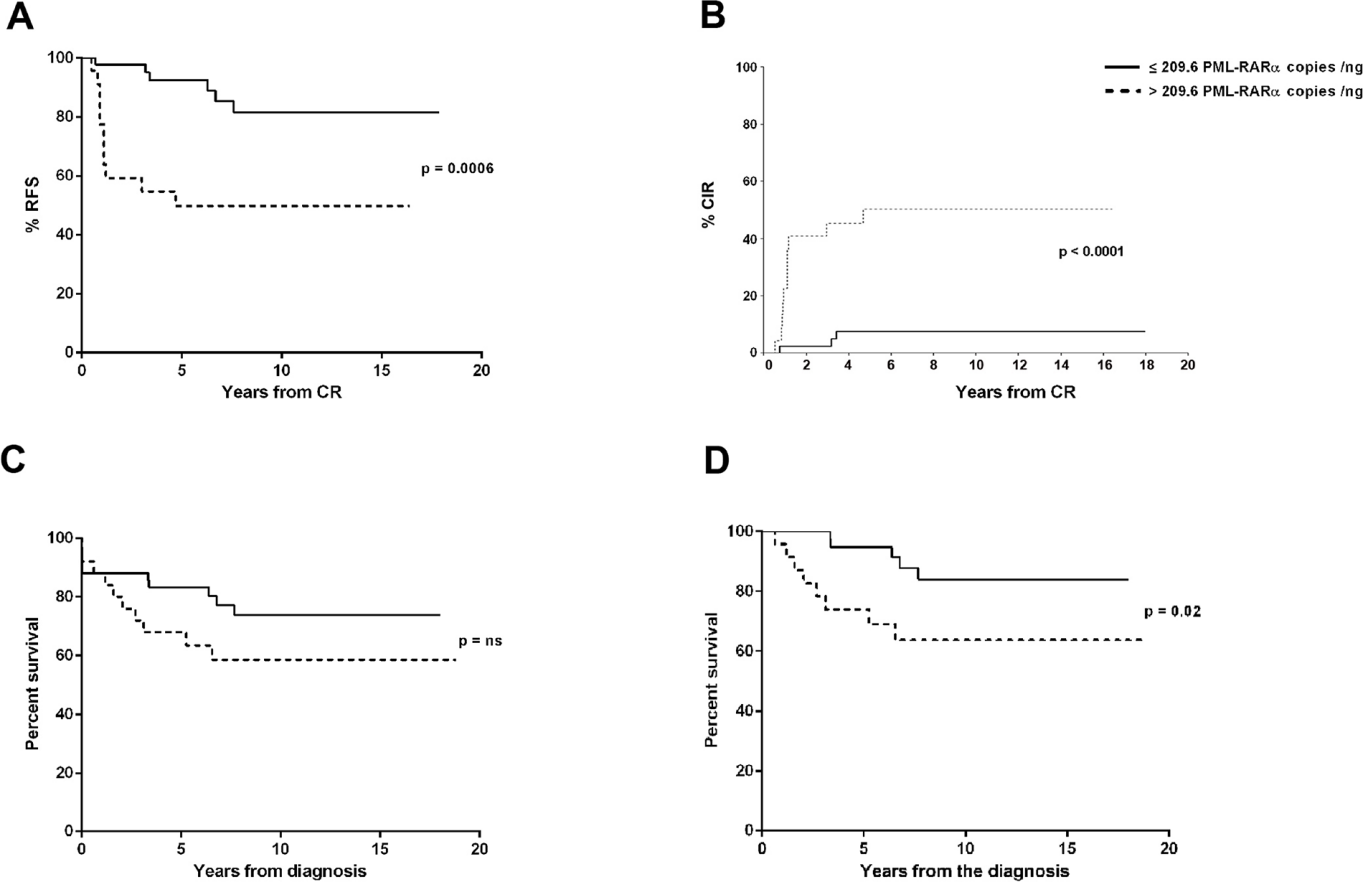
RFS, CIR and OS stratified by pretreatment *PML-RARA* copies/ng RFS probability **A.** and CIR **B.** according to the *PML-RARA* transcript value at diagnosis. OS analysis of the entire cohort **C.**, and after excluding the ED group from the analysis **D.**.

ddPCR experiments were successfully performed in all 76 patients. The median concentration of the fusion transcript was 124 *PML-RARA*/ng (min. 7.76 – max 1070) (Figure 1). In our series, 14 patients suffered disease recurrence (12 and 2 patients with molecular and hematological relapse, respectively) with a median time to relapse of 1.16 years (min. 0.58 – max 3.83 years). The amount of *PML-RARA* transcript at diagnosis in the group of patients who relapsed was higher than in that with continuous complete remission (CCR) (272 vs 89.2 *PML-RARA*/ng, *p* = 0.0004, respectively). The same statistically significant difference was observed when comparing patients who relapsed with those belonging to the early death (ED) group (272 vs 110 *PML-RARA*/ng, *p* = 0.04, respectively) (Figure 2). Moreover, considering the arbitrary cut-off of 124 *PML-RARA*/ng (the median value of our APL series), a higher proportion of patients who relapsed (85.7%) had >124 *PML-RARA*/ng compared to the CCR group (39.6%) (odds ratio 0.10; *p* = 0.002). Further parameters (age, sex, WBC count, M3/M3v, bcr transcript type, FLT3 mutation status, CD34 and CD2 expression, relapse risk score) were assessed to verify the presence of different amounts of the fusion gene transcript at diagnosis among these different categories but yielded no statistically significant differences (data not shown). Using receiver operating characteristic (ROC) analysis, the optimal *PML-RARA* concentration threshold for discriminating between outcomes (CCR versus relapse) was estimated as 209.6 *PML-RARA*/ng (area under the curve [AUC], 0.78; 95% CI, 0.68–0.89, *p* < 0.0001) (Figure 3). Eleven (79%) and 40 (75.4%) patients belonging to the relapsed and CCR groups had values above and below this threshold, respectively. Among the 67 APL patients who achieved complete remission (CR) after the induction treatment, those with >209.6 *PML-RARA*/ng had a worse relapse-free survival (RFS) compared to those with ≤209.6 *PML-RARA*/ng (4.7 vs median not reached, *p* = 0.0006) (Figure 4A). At 5-year follow-up, patients with >209.6 *PML-RARA*/ng had a cumulative incidence of relapse (CIR) of 50.3% (95% C.I. 33.1–76.4%) whereas 7.5% (95% C.I. 2.5 – 22.5%) of the patients with ≤209.6 *PML-RARA*/ng suffered a relapse (*p* < 0.0001) (Figure 4B). There was no difference in terms of overall survival (OS) between patients with ≤209.6 *PML-RARA*/ng and those with >209.6 *PML-RARA*/ng (Figure 4C). It is noteworthy that when the group of patients with ED was excluded from the OS analysis, the difference between the two groups was statistically significant (median not reached for both groups, *p* = 0.02) (Figure 4D). There was no difference in the time required to achieve CR between APL patients with more or less than 209.6 *PML-RARA*/ng at diagnosis. Multivariate analysis by Cox proportional hazards regression model was performed for RFS after including all candidate variables (Age [<60 vs >60 y.rs], FLT3 mutation status [FLT3-ITD vs FLT3wt/D835], Sanz's score [high vs low/intermediate], *PML-RARA* concentration [≤209.6 copies/ng vs >209.6 copies/ng], bcr transcript type [1–2 vs 3]). The relapse hazard was 9 times higher for patients with *PML-RARA* concentration >209.6 copies/ng (HR 9.26, 95% CI 2.47–34.6, *p* = 0.0009) than for patients with <209.6 copies/ng. In the model, the *PML-RARA* molecular burden before treatment was identified as the sole independent prognostic factor for APL relapse (Table 2).

## DISCUSSION

To date, the pretreatment amount of the *PML-RARA* transcript has not been considered as a prognostic factor in APL. This conclusion was based on a study that quantified the chimeric transcript by RT-qPCR at diagnosis [16]. In addition, the pretreatment concentration of the *PML-RARA* transcript, together with the concentration after consolidation treatment, has been considered to assess the prognostic impact of the clearance kinetics of the fusion transcript [11, 20–22]. Only one report showed that the *PML-RARA* copy number may be related to the patient's relapse risk [22] but this finding was never confirmed by other groups. In our study, we employed ddPCR technology which allows easy measurement of the absolute copies number of the *PML-RARA* transcript. Our data show that, in APL, the *PML-RARA* burden before induction therapy is an important prognostic factor, able to identify patients at higher risk of relapse and with a worse RFS. Indeed, we found that the prognostic performance of the pretreatment *PML-RARA* transcript concentration is better than that of the relapse risk score, which is usually employed to define the degree of APL recurrence risk; in fact, among the 14 APL patients who relapsed, only 4 (28.5%) were high-risk according to Sanz's score whereas 11 (78.5%) had a *PML-RARA* molecular burden at diagnosis of >209.6 copies/ng. The threshold of 209.6 *PML-RARA* copies/ng identified by our analysis could identify patients at higher risk of relapse, but produced 12 (22.6%) “false positives” within the CCR patients group. In this regard, it should be noted that among these 12 APL patients, 1 had a short follow-up (7 months) and 5 were included in the AIDA-2000 protocol, where, unlike the AIDA-0493 regimen, all patients received ATRA for 15 days during the three cycles of consolidation. Therefore, one might speculate that in these patients the predictive ability of the molecular test may have been affected by a too short follow-up for the relapse to appear, or by the introduction of ATRA in consolidation therapy. Regarding this latter point it could be added that in the group of patients who relapsed there were three “true positives”, included in the AIDA-2000 regimen, who showed relapse at the end of the consolidation therapy. Therefore, the possibility that the molecular test produced “false positives” just because the biological risk was corrected by therapeutic intervention remains a mere speculation.

The OS rate of APL is affected mostly by two factors: the rate of relapse, and the high frequency of ED. In fact, with the current treatment approaches, up to 20% of relapses have been reported in several studies, by far the most common treatment failure in APL being ED. While several pretreatment prognostic factors have been reported to be associated with ED, such as older age, FLT3-ITD mutation status, WBC and platelet count, and LEF1 gene expression [23–26], Sanz's score [10] remains the sole prognostic index employed in clinical practice to identify the patient's relapse risk, although this finding could not be confirmed in a large German study [27]. Our data clearly show that the *PML-RARA* molecular burden before treatment is not linked to ED. In fact, the median copy number of the *PML-RARA* transcript in this group was not different to that observed in the entire APL series. Moreover, among the 9 ED, 7 (77.7%) had a *PML-RARA* ≤209.6 copies/ng, as did most of the patients belonging to the CCR group. For this reason, after excluding the ED group from the OS analysis, it can be seen that the *PML-RARA* molecular burden at diagnosis also has a prognostic impact on survival.

In conclusion, our study shows that the ddPCR technology represents a powerful tool to assess the presence of *PML-RARA* transcript at diagnosis as well as to define the relapse risk; in fact, the pretreatment *PML-RARA* transcript concentration is a robust independent prognostic factor for the identification of APL patients at higher risk of relapse, and could therefore be used to improve risk stratification in order to develop more individualized treatment regimens for high-risk APL patients. Moreover, in our opinion, it is possible that in the very near future ddPCR may also become a valuable tool for monitoring MRD in APL.

## METHODS

### Patients

One hundred and four consecutive patients with newly diagnosed APL were observed and treated with the AIDA-0493 [28] and AIDA-2000 [29] GIMEMA group protocols at the Hematology Section, Bari University Hospital, between January 1996 and December 2013. The diagnosis was initially morphological and was confirmed in all cases by detection of the *PML-RARA* fusion gene, as reported [30]. All analyzed patients showed >50% of promyelocytic leukemic cells in bone marrow aspirate. *PML-RARA* expression analysis by ddPCR was performed in 76 patients with sufficient available material (median age 46 years, range 16 to 88 years; 35 males and 41 females). The median follow-up time was 6.6 years for the entire cohort. The main characteristics of the patients are reported in Table 1. All treatments were administered in accordance with the Declaration of Helsinki and approved by the institutional local review board, and all patients provided written informed consent. All 76 patients started induction treatment but 9 (11.8%) died within 30 days of hospital admission (4 of them before definitive therapy could be instituted): 7 (9.2%) patients due to hemorrhagic/infective complications and 2 (2.6%) patients due to the differentiation syndrome. These patients represent the ED group.

**Table 1:** APL patients characteristics

**Sex**	
*Male*	35 (46%)
*Female*	41(54%)
**Age**	
< *60 years*	59(77%)
≥ *60 years*	17(23%)
**FAB**	
*M3*	71 (93%)
*M3v*	5 (7%)
**bcr transcript type**	
*bcr1*	37 (49%)
*bcr2*	5 (6%)
*bcr3*	34 (45%)
**WBC count (× 10^3^/L)**	
≤*10.0*	53 (70%)
>*10.0*	23 (30%)
**CD34 expression**	
*CD34+*	23 (30%)
*CD34-*	54 (70%)
**CD2 expression**	
*CD2+*	17 (22%)
*CD2-*	59 (78%)
**FLT3 molecular status**	
*ITD+*	19 (25%)
*D835+*	12 (16%)
*WT*	45 (59%)
**Sanz's score**	
*Low*	15 (20%)
*Intermediate*	40 (53%)
*High*	21 (27%)
**Regimen treatment**	
*AIDA-0493*	31 (46%)
*AIDA-2000*	36 (54%)
**Outcome**	
*ED*	9 (12%)
*CCR*	53 (70%)
*REL*	14 (18%)

Clinical and biological characteristics of the 76 APL patients included in the study.

**Table 2:** Multivariable Cox model analysis for RFS

Variabile	HR	95% CI	*p* value
FLT3 ITD: positive vs negative	0.3	0.07–1.58	0.16
Sanz's score: H vs L-I	2.34	0.57–9.59	0.23
PML-RARa copies/ng: >209.6 vs <209.6	9.26	2.47–34.6	0.0009
Age (years) >60 vs ≤60	1.06	0.28–3.95	0.93
bcr transcript type: bcr3 vs bcr1/2	2.59	0.73–9.23	0.14

Multivariate analysis according to the Cox proportional hazards model for RFS. Results obtained in 67 APL patients who achieved CR. HR indicates the hazard ratio; CI, the confidence interval. HRs >1 or <1 indicate an increased or decreased risk, respectively, of an event for the first listed category.

### Molecular analysis

The RNA concentration was assessed using a Qubit 2.0 Fluorometer (Life Technologies). Total RNA derived from bone marrow (BM) cells at APL diagnosis was reverse transcribed into cDNA using the QuantiTect reverse transcription kit (Qiagen, Chatsworth, CA, USA). Absolute quantification of *PML-RARA* transcript was performed by ddPCR analysis, a novel PCR technology that allows a highly reproducible absolute quantification of input nucleic acid molecules [18]. In fact, compared to RT-qPCR, the digital PCR approach offers a greater precision and reproducibility, as well as the capability to obtain an absolute quantification without external references and robustness to variations in PCR efficiency [17]. Moreover, in ddPCR it is possible to use the same primers and probes as RT-qPCR with higher sensitivity and precision [19]. In our study, ddPCR experiments were performed using primers and probes for *PML-RARA* bcr1, bcr2, and bcr3 isoforms previously described [31]. *ABL1* was used as control gene to confirm the good quality of cDNA samples; the *PML-RARA* and *ABL1* transcripts were tested in multiplex in the same well. Bio-Rad's QX200 ddPCR system combines water-oil emulsion droplet technology with microfluidics. Each sample is partitioned into 20, 000 droplets by a droplet generator and each droplet is amplified by PCR. Then, droplets are streamed in single file on a droplet reader, which counts the fluorescent positive and negative droplets to define the target concentration. The *PML-RARA* primers and probes were at final concentrations of 900 and 250 nmol/L, and 50 ng of cDNA template was used in a final volume of 20 uL. The 20-uL ddPCR reaction mixture was then loaded into the Bio-Rad DG8 droplet generator cartridge. A volume of 70 uL of droplet generation oil was loaded for each sample. The cartridge was placed into the QX200 droplet generator. The generated droplets were transferred to an Eppendorf 96-well PCR plate (Eppendorf, Hamburg, Germany). The plate was sealed with a BioRad pierceable foil heat seal, and samples amplified on the T100 BioRad thermal cycler. Thermal-cycling conditions were 95°C 10 minutes (1 cycle), 94°C 30 seconds (ramp rate 2°C/second, 40 cycles), 60°C 1 minute (ramp rate 2°C/second, 40 cycles), 98°C 10 minutes (1 cycle), and 4°C hold. After amplification, the 96-well PCR plate was loaded on Bio-Rad QX200 droplet reader and ddPCR data were analyzed with QuantaSoft analysis software (version 1.7.4). Target concentration in each sample was expressed as *PML-RARA* copies/ng. Moreover, FLT3 (ITD and TKD) mutations were investigated on total BM RNA by allele specific oligonucleotide (ASO)-PCR and PCR followed by enzymatic digestion [32–33].

### Flow cytometry analysis

Leukemic cell analysis was performed on BM cells by standard methods using monoclonal antibodies directed against CD2 and CD34 (Becton Dickinson, Milan, Italy). Flow cytometric analysis was performed on a FACSCantoTM II flow cytometer (Becton Dickinson Immunocytometry System, Mountain View, CA, USA). A sample was considered antigen-positive if ≥ 20% of the leukemic cells reacted with a particular monoclonal antibody.

### Statistical analysis

Clinical and biological features between groups were compared using Fisher's exact test for categorical data and nonparametric Mann-Whitney U test for continuous variables. A *p* value < 0.05 was considered significant. ROC curves were generated using the XLSTAT version 2014.5.03 (AddinsoftTM). Optimal thresholds along the ROC curves were calculated using the Youden index; the AUC significantly different from 0.5 means that the test is very powerful [34]. Survival curves were calculated by the Kaplan-Meier method with log-rank comparing differences between survival curves. OS endpoints, measured from the date of diagnosis, were dead or alive at last follow-up. RFS was counted from the achievement of documented CR until APL relapse. The CIR was estimated with the use of the proper nonparametric estimator, and between-group comparisons were performed with Gray's K-sample test [35–36]. Multivariable Cox proportional hazards models were used to study factors associated with RFS as categorical variables (Age [<60 vs >60 y.rs], FLT3 mutation status [FLT3-ITD vs FLT3wt/D835], relapse risk grade [high vs low/intermediate], *PML-RARA*/ng [≤209.6 copies/ng vs >209.6 copies/ng], bcr transcript type [1–2 vs 3]); no variable selection technique was used, and all variables remained in the multivariable model.
